# [Retracted] Effects of miRNA-455 on cardiac hypertrophy induced by pressure overload

**DOI:** 10.3892/ijmm.2026.5946

**Published:** 2026-08-03

**Authors:** Chuntao Wu, Shimin Dong, Yongjun Li

Int J Mol Med 35: 893-900, 2015; DOI: 10.3892/ijmm.2015.2105

Following the publication of this paper, it was drawn to the Editor's attention by a concerned reader that, in addition to a pair of data panels showing images of myocardial fibrosis in Fig. 5A on p. 897 having an overlapping section, western blots data featured in Fig. 6B for the '2 weeks' experiments on p. 898 were strikingly similar to data that had already been submitted to the journal *PLoS One* in a paper that was written by different authors at different research institutes.

Given that the abovementioned data had already been submitted for publication before the receipt of this paper at *International Journal of Molecular Medicine*, the Editor has decided that it should be retracted from the Journal. The authors were asked for an explanation to account for these concerns, but the Editorial Office did not receive a reply. The Editor apologizes to the readership for any inconvenience caused.

